# Comparative analysis between operative and non-operative acetabular labral tear injuries in division 1 collegiate athletes

**DOI:** 10.1038/s41598-023-36454-0

**Published:** 2023-06-10

**Authors:** Scott S. Hyland, Andres D. Maeso, Mark Rogers, Mike Goforth, P. Gunnar Brolinson

**Affiliations:** 1 Ohio Health Doctors Hospital, 5100 W Broad St, Columbus, OH 43228 USA; 2 Edward Via College of Osteopathic Medicine, Blacksburg, USA; 3 Virginia Tech Sports Medicine, 2265 Kraft Dr SW, Blacksburg, VA 24060 USA

**Keywords:** Musculoskeletal system, Orthopaedics

## Abstract

Acetabular labral tears have shown to be difficult to diagnose and manage in an active and competitive athletic population. The goal of this study was to compare NCAA Division 1 collegiate athletes undergoing operative and non-operative management of their labral injuries by assessing ability to return to competition and secondarily evaluate days lost from sport. A retrospective cohort analysis was conducted on Division 1 collegiate athletes from 2005 to 2020, incorporating all varsity university sports. Records showing MRI confirmed diagnosis were included in the cohort, as well as all pertinent clinical data. Data revealed 10/18 (55%) of individuals managed conservatively versus 23/29 (79%) surgically (*p*-value = 0.0834) were able to return to sport following treatment. Of those athletes, 22 surgical patients experienced a mean of 324 days ± 223 days lost from sport and nine conservatively managed patients experienced a mean of 27 days ± 70 lost days (*p*-value < 0.001) Seven of nine conservatively managed patients were able to continue competition while undergoing treatment. Findings suggest no statistical significance regarding operative vs non-operative management of acetabular labral tears. The majority of athletes returning to sport and treated conservatively were able to resume competition during treatment. Therefore, treatment of these injuries should be individualized based on athlete’s symptoms.

## Introduction

The acetabular labrum in the hip serves several important functions such as shock absorption, joint lubrication, pressure distribution, and providing stability to the joint surface. Labral tear etiology has been shown to be secondary most commonly to acetabular dysplasia; however, femoral acetabular impingement (FAI), trauma, hypermobility, and degeneration of the hip have also been found to be causes^1–3^. McCarthy and Lee showed that patients with mild-to-moderate hip dysplasia and hip pain demonstrated 72% of 170 hips studied had labral tears, 93% of which were located anterior region of labrum^4^. Wenger also demonstrated that 87% of hips with labral tears studied revealed at least one abnormal radiographic finding, while 13% had no radiographic structural abnormality^5^.

Typically, the anterosuperior aspect of the labrum is involved with a higher incidence found in physically active females^3^. Such injuries can be problematic, particularly for athletes needing to return to competition at an elite level for their respective sport primarily due to pain and mechanical symptoms. Symptoms may include groin pain or mechanical symptoms such as clicking, locking, or catching. Several treatment approaches have been studied in order to determine how to effectively manage these injuries in athletes competing at a high level. However, acetabular labral tears have shown to be difficult to diagnose and manage in an active and competitive athletic population.

Initially, physical therapy combined with relative rest, NSAIDs, and corticosteroid injections have been recommended; however, arthroscopic surgical treatment has become the gold standard in treating labral tears of the hip^6–8^. Several surgical techniques have been described, including but not limited to labral debridement, primary labral repair, labral reconstruction with autograft or allograft in a primary versus staged approach, predominantly conducted with standard or minimally invasive arthroscopic approaches^9–12^.

Several studies have demonstrated athletes’ clinical improvement and ability to return to competition following surgical management with proper post-operative therapeutic protocol^7,8,13^.

There is a paucity of quantified data comparing athletes managed operatively versus non-operatively and the authors of this study look to provide further insight into these patients. Limitations of past studies include the number of subjects and the various therapeutic regimens, thus making it difficult to compare recovery courses and overall prognosis. The aim of this study was to perform a retrospective data analysis comparing NCAA Division 1 collegiate athletes at a single institution undergoing operative and non-operative management of their acetabular labral injuries. Our hypothesis was that individuals undergoing surgical management of their labral pathology would have a higher probability of returning to their respective sport at pre-injury performance levels in comparison with those treated with non-operative measures only.

## Methods

Following institutional review board approval, a retrospective cohort analysis was conducted on athletes treated for acetabular labral tears and their clinical courses of recovery. Primary outcomes of interest included performance/participation level defined as return to current level of competition per sport. Time to return to play was measured as days until next game competition was also documented.

Subjects included in this study participated in NCAA division 1 athletics at Virginia Polytechnic Institute and State University (VT). Both male and female patients, regardless of age, were included if they were categorized as student athletes in the electronic medical records (EMR) at Virginia Tech (2005–2020). The study included all major sports at the university (Table 1). A total of 47 patients were included in this study. All patients included in the cohort had a confirmatory MRI diagnosis of a hip labral tear as confirmed by musculoskeletal radiologist or included in the discussion of the primary surgeon. Specific location, characterization, or extent of the tear was not included for every patient and, therefore, was not evaluated as part of this study. This was a convenience sample selected based on the number of clinical cases available through the VT athletic medical records, which negated the need for exclusion criteria in this analysis. Athletes self-selected operative vs non-operative management based on a number of factors including in-season vs out of season injury, ability to participate in sport at a high-performance level, class year and eligibility (e.g. freshman vs senior) as well as other personal reasons. These results were not provided to subjects nor was any personal identifying information included in the study. All data is de-identified and was reported in aggregate fashion only. High-performance level of competition was defined in this study as participants being able to participate in game competition of their individual sporting event.

**Table 1:** Demographic and sport specific analysis of cohort.

Characteristic	Overall
Age	20.2
Male	23
Female	24
Sports of participants	
Football	8
Cross Country	6
Lacrosse	5
Basketball	5
Soccer	4
Volleyball	4

All 22 sports were included, however, those less than 4 participants in the study per sport were not included in the table.

Records were reviewed using a keyword search for acetabular labral injuries, tears, and hip impingement including their respective rehabilitation processes and outcomes. All methods were performed in accordance with the relevant guidelines and regulations, and all identifying patient information was not included within the contents of this manuscript. Written informed consent was waived by the ethics committee of Virginia Tech as this was a retrospective chart review of patient data. Data was then collected and stored in a secure password-protected database (Microsoft Excel). Gender, age, sport, incident date, MRI impression, date of MRI, treatment (operative or non-operative), surgery date, and date of return to sport was included for each patient. Follow up intervals as well as discharge criteria outside of return to play and days lost from sport were not provided given the retrospective nature of this study.

After data collection, statistics were analyzed with means and standard deviations and compared using Student’s t-tests. Frequencies were calculated for continuous variables and compared using Pearson Chi-Square test for increased accuracy in small proportion analysis. A significance level of *p* < 0.05 was set prior to investigation.

## Results

Forty-seven Division 1 college athletes were included in this retrospective cohort analysis to assess return to play within their respective sport following operative vs non-operative management of hip labral pathology. Twenty-nine individuals underwent operative treatment and eighteen were treated non-operatively with anti-inflammatory medications, physical therapy and clinically controlled sport-specific rehabilitative protocols. Twenty-three of twenty-nine patients treated operatively (79%) ultimately returned to high level function in their respective sport, while 10 of 18 patients treated non-operatively (55%) were able to return to high level competitive play. Although operative treatment resulted in a clinically higher percentage of return to high level competition, this was not found to be statistically significant using Pearson Chi-Square test of the displayed variables seen in Table 1.

Secondary outcome evaluation of the data compared days lost from sport between the surgical and non-operative arms. Days lost from sport involved the amount of time, in days, the athlete was held from competition at index injury until clearance for game competition. Nine patients treated non-operatively lost an average of 27 days of sport during recovery compared to 22 surgically managed patients who lost an average of 324 days prior returning to competitive athletics (*p* < 0.001). Seven of nine non-operatively managed patients were able to continue to compete during their rehabilitation protocol. One patient from both the operative and non-operative cohort who returned to sport had unclear data as to days lost from sport, therefore, they were not included in the calculated means to avoid misrepresentation of data.

## Discussion

The primary outcome analyzed in this study was to evaluate athletes’ ability to return to competition at the Division 1 level after non-operative versus operative management of hip labral pathology. Our findings did suggest a clinical trend in participants returning to pre-injury levels of competition in the operative arm (79% vs. 55%; Table 2), however, the results did not reach statistical significance (*p*-value = 0.0834). There is debate regarding the effectiveness of the operative approach for college athletes, but previous literature has demonstrated good short-term relief with hip arthroscopy for internal hip joint labral pathology^7,14–16^. Weber et al. demonstrated that nearly 90% of athletes returned to sport following hip arthroscopies for labral pathology associated with femoral acetabular impingement, with the only difference in return-to-sport rate being a lower rate of return in endurance athletes (66%; *p*-value < 0.001)^17^. Maldonado et al. also compared a wider range of athletic population levels (recreational, high school, collegiate) and demonstrated a 78% return to sport within 1 year of surgery^18^. Cianci et al. describe a subgroup of their patient population of nearly 25% that were able to participate in athletic competition with non-operative therapeutic methods including intra-articular injection, physical therapy, or combination of the two^19^. The patient cohort in this study suggests a higher percentage of individuals returning to sport with non-operative management compared to other studies, which may be multifactorial in nature. This may be explained by the short-term improvement from non-operative protocol which may have provided enough relief to participate in athletic competition for the remainder of eligibility. This also could vary due to sport specific demand which was not evaluated as part of this study. Non-operative treatment in patients with labral tears has limited investigation, and the results of this study propagate the necessity for further investigation.

**Table 2:** Surgical versus conservative management in athletes with hip labral pathology.

Return to play—Surgical versus conservative management				
Management	Returned to play		Total	*P*-value
	Yes	No		
Surgical	23 (79%)	6 (21%)	29	0.0834
Conservative	10 (55%)	8 (44%)	18	
			47	

Our secondary evaluation of lost days of sport revealed statistical significance in comparison of operative and non-operative arms (*p*-value < 0.001; Table 3). The surgical cohort return to competition (324 ± 223 days) was consistent with Weber et al. (1.96 ± 0.94 years)^17^. Of the nine non-operatively managed athletes that were ultimately able to return to their individual sport, seven of them continued competitive play during their rehabilitation protocol. Surgical intervention has historically been superior to non-operative management in athletes with labral tears, however, it is compelling that a large portion of non-operatively managed athletes demonstrated ability to compete successfully with their injury using a non-operative management approach. This suggests that a comprehensive non-operative management approach (see results section above for therapeutic approach) may allow an athlete to finish their current season, but does not negate the possibility of future surgical intervention. Most of the patients of the surgical group were initially treated non-operatively prior to imaging confirmed diagnosis and ultimately converted to surgical management after failing conservative measures. Failure of conservative management was not explicitly defined besides the lack of improvement necessitating surgical intervention.

**Table 3:** Cohort analysis to assess days of lost play in their respective sport following operative versus non-operative management of hip labral pathology.

Lost days of sport—Surgical versus conservative management				
Management	n	Mean	Standard Deviation	T-test *P*-Value
Surgical	22	324	223	< 0.001
Conservative	9	27	70	

Most conservatively managed subjects (7/9) continued to participate despite being clinically diagnosed with a labral tear. There was a single individual from both surgical and non-operative cohort with unclear documented lost days of sport and therefore, was not included in these calculations to avoid skewed results.

It is the authors’ opinion to correlate treatment based on symptoms, with more aggressive measures reserved for increased severity. There needs to be a complete work up to ensure labral tear is the etiology of groin pain as there is considerable evidence suggesting 50–60% of asymptomatic labral tears may be present in the competitive athlete^20^. This may assist physicians providing information to the athlete to make an informed decision taking into account their symptoms, preferences, collegiate eligibility, and future athletic aspirations.

This study is subject to the limitations of a retrospective chart review. Our study consisted of a limited number of participants, with lack of documented consistent follow up intervals, and discharge criteria which limited the secondary subset analysis. Stricter adherence to follow up would provide a more accurate assessment in both operative and non-operative arms. MRI findings were also limited in attainability, and the review restricted the authors to relying on radiology interpretation as well as primary surgeon treatment in these athletes. More descriptive, objective characterization of the labral pathology could have provided clinical relevance in understanding treatment methodology and outcomes. Also, all Division 1 sports at VT were included in this study, and a more accurate depiction may have been attained with sport-specific analysis since the biomechanical demands of different sports will affect hip performance in student-athletes uniquely. This was demonstrated in Weber et al. with endurance athletes. Further delineation of sport-specific treatment trends may ultimately help orthopedic providers advise with more predictable outcomes for their patients, and is recommended in future analyses of college athlete populations undergoing treatment for hip labral tears. In season versus out of season injury and treatment could have also influenced primary and secondary outcomes which would be another benefit from evaluation of athletes on a sport specific basis. Patient decision making to proceed with surgery or non-operative management could also have played a role for multiple reasons such as eligibility remaining for competition, pain severity, or continued competitive sport specific pursuits. This would serve as valuable information in future prospective or retrospective literature to assess personal patient factors and ultimate definitive labral pathology management. Finally, all hip arthroscopy procedures were included (debridement vs. repairs), which may have influenced either our primary or secondary outcomes. Further investigation of the impact of repair or debridement specifically may be valuable in assessing influence on return to high level of competition in college athletes. With this in mind, alterations in conservative treatment protocol also could theoretically positively or negatively impact our primary and secondary outcomes. Variability in operative or conservative treatments are inherently difficult to control as seen in prior retrospective studies mentioned.

A power calculation for a future prospective trial was also conducted. Based upon the proportions observed within this study (79% achieving returning to pre-injury levels of competition in the operatively treated group, 55% achieving return to pre-injury levels of competition in the non-operatively treated group), we calculate 57 subjects per group are required to achieve 80% statistical power to declare the difference in proportions statistically significant using a two tailed Type I error rate of 0.05. We submit that if our patients met the number of individuals in the power analysis, there may be a higher likelihood for a statistically significant outcome favoring operative management, however, the current data does not support this conclusion. Furthermore, a future prospective trial will allow further examinations of which clinical factors are associated with the choice of non-operative treatment and the success of non-operative treatment.

In conclusion, there is still significant debate regarding the management of acetabular labral tears in collegiate athletes and their ability to return to Division 1 competition. Risks and benefits of surgery should be weighed, and complications considered prior to advancing with operative measures. Complications seen in hip arthroscopy include infection, deep vein thrombosis, nerve injury (pudendal and lateral femoral cutaneous nerve most commonly secondary to traction and portal placement), incomplete repair, and swelling^21^. Comprehensive non-operative management should be pursued prior to consideration of surgical intervention, as a considerable number of athletes have demonstrated the ability to continue to compete successfully during rehabilitation. Therefore, it is imperative that the treating physician appropriately guide their athlete through this decision-making process to optimize outcomes. Although historical data suggests that surgical intervention is superior to non-operative management, these current findings suggests compelling findings that provide a different perspective. Regardless of treatment method, management of these injuries should continue to be investigated further and we recommend an individualized approach to the intervention of each patient.

## Data Availability

The datasets generated and/or analyzed during the current study are available from the corresponding author on reasonable request.
